# Correction: Advanced design and Engi-economical evaluation of an automatic sugarcane seed cutting machine based RGB color sensor

**DOI:** 10.1371/journal.pone.0324915

**Published:** 2025-05-19

**Authors:** Abdallah Elshawadfy Elwakeel, Loai S. Nasrat, Mohamed Elshahat Badawy, I. M. Elzein, Mohamed Metwally Mahmoud, Kitmo, Mahmoud M. Hussein, Hany S. Hussein, Tamer M. El-Messery, Claude Nyambe, Salah Elsayed, Manar A. Ourapi

The affiliation for the eighth author is incorrect. The correct affiliation is not indicated. Hany S. Hussein is not affiliated with #5 but with: Electrical Engineering Department, College of Engineering, King Khalid University, Abha 62529, Saudi Arabia
