# Supplementary material for: Advanced design and Engi-economical evaluation of an automatic sugarcane seed cutting machine based RGB color sensor
Source: PLoS One. 2024 Oct 17;19(10):e0306584. doi: 10.1371/journal.pone.0306584 (PMC11486418; doi:10.1371/journal.pone.0306584)
Supplement: Attachment — Submitted filename: Response to reviewers.docx [file pone.0306584.s001.docx]

****Technical response to the reviewers****	May 23^th^, 2024

**Journal name: PLOS ONE**

**No.: PONE-D-24-12311**

**OLD Title:** “**~~Advanced Design and Engineering Economical Evaluation of a Low-Cost Automatic Sugarcane Seed Cutting Machine Based RGB Color Sensor and IoT Technology~~”**

**NEW Title: “Advanced Design and Engi-Economical Evaluation of an Automatic Sugar-cane Seed Cutting Machine Based RGB Color Sensor”**

**Abdallah Elshawadfy Elwakeel^1^, Loai S. Nasrat^2^, Mohamed Elshahat Badawy^3^, I. M. Elzein^4^, Mohamed Metwally Mahmoud^5^, Kitmo^6&*^, Mahmoud M. Hussein^5,7^,** **Tamer M. El-Messery ^8^, Claude Nyambe^8^, Salah Elsayed^9^ and Manar A. Ourapi^1^**

^1^ Agricultural Engineering Department, Faculty of Agriculture and Natural Resources, Aswan University, Aswan 81528, Egypt; [Abdallah_elshawadfy@agr.aswu.edu.eg](mailto:Abdallah_elshawadfy@agr.aswu.edu.eg), [Manarourpi@gmail.com](mailto:Manarourpi@gmail.com)

^2^ Electrical Power Engineering Department, Faculty of Engineering, Aswan University, Aswan 81528, Egypt, [loaisaad@yahoo.com](mailto:loaisaad@yahoo.com)

^3^ Agricultural Engineering Research Institute - Dokki – Giza 12611, Egypt, [Mohamedelshahat@gmail.com](mailto:Mohamedelshahat@gmail.com)

^4^Department of Electrical engineering, University of Doha for Science and Technology, Doha, Qatar

60101973@udst.edu.qa

^5^Electrical Engineering Department, Faculty of Energy Engineering, Aswan University, Aswan 81528, Egypt, [Metwally_M@aswu.edu.eg](mailto:Metwally_M@aswu.edu.eg)

^6&*^University of Maroua, National Advanced School of Engineering of Maroua, Department of Renewable Energy, P.O. Box 46 Maroua, Cameroon, [kitmobahn@gmail.com](mailto:kitmobahn@gmail.com)

^7^Department of Communications Technology Engineering, Technical College, Imam Ja’afar Al-Sadiq University, Baghdad, 10053, Iraq, mahmoud_hussein@aswu.edu.eg

^8^International Research Centre “Biotechnologies of the Third Millennium”, Faculty of Biotechnologies (BioTech), ITMO University, St. Petersburg, 191002, Russia, [telmesseri@itmo.ru](mailto:telmesseri@itmo.ru); [Clauderahl01@gmail.com](mailto:Clauderahl01@gmail.com)

^9^Agricultural Engineering, Evaluation of Natural Resources Department, Environmental Studies and Research Institute, University of Sadat City, Sadat City 32897, Egypt; [salah.emam@esri.usc.edu.eg](mailto:salah.emam@esri.usc.edu.eg)

*Corresponding author: [kitmobahn@gmail.com](mailto:kitmobahn@gmail.com)

**Dear Editors and Reviewers**

The authors are thankful to the learned Editors and Reviewers for their thoughtful and detailed comments to improve the quality of the manuscript. The authors have tried to address all the concerns, and the corrections are incorporated in the revised manuscript. The replies to the reviewer’s comments are provided below.

We hope that this revised version can meet the reviewer’s expectations and the standards for publication in **PLOS ONE Journal.**

The changes incorporated in the revised manuscript are highlighted in YELLOW.

**Editor's Comments:**

Our sincere thanks and appreciation to the Editors for recommending the submission of the revised manuscript with major revision. To improve the quality of the manuscript, the reviewer's queries are addressed, and their suggestions are incorporated into the revised manuscript.

**Reviewer Comments:**

**Reviewer 1:**

**Comments to the Authors:**

**Comment**:** The manuscript titled “Advanced Design and Engineering Economical Evaluation of a Low-Cost Automatic Sugarcane Seed Cutting Machine Based RGB Color Sensor and IoT Technology” is adequately structured, with an introduction that highlights the importance and the problem of study very well justified, although, the theoretical framework could be updated. The objectives are clear, demonstrated in the development of the study and in the results and conclusions obtained. The figures and tables presented are sufficient to characterize the experimental development, as well as to demonstrate the results obtained.

**Authors response**: The authors are thankful to the honorable reviewer for the words of encouragement and trust in our work.**

**Comments1:  *Justify the experimental choices and variables evaluated in the experiments (methodology).***

**Response1:** The authors are extremely thankful to the reviewer for this thoughtful point. We agree with you, and we corrected it, kindly check the updated paper (***titles 2.4 & 2.5***).

**Comment 2:  *Although the economic analysis is important, it is limited to the region where the study was developed. Could this study be replicated to other sugarcane producing regions? In addition to the technology evaluated, the other evaluations seem to me to be more of a case study, applied to the region. Check the importance and meaning of the economic evaluation in the manuscript.***

**Response 2:** The authors are extremely thankful to the reviewer for this thoughtful point. We agree with you, and we added it, kindly check the updated paper (***title 3.5 & Table 4***). Where due to the lack of economic studies for the automatic sugarcane seed cutting systems, it is impossible to determine the economic efficiency of the manufactured machine in comparison to other systems worldwide. So, this study aimed to provide an economic analysis of the machine, which can be used as a reference point for evaluating and comparing the automatic sugarcane seed cutting systems in the future. On the other hand, for both farms and manufacturers, understanding the economics of owning and operating this machine is crucial.

Absolutely, this study can be replicated in any region, where all assumptions are shown in materials and methods and highlighted in yellow.

**Comment 3**:  ***Expand the discussion with recent studies. The discussion is succinct and vague in relation to the good results obtained.***

**Response 3:** The authors are extremely thankful to the reviewer for this thoughtful point. We agree with you, and we added it, kindly check the updated paper (***Results and Discussion*** ***section***).

**Comment 4:**  The conclusions are very extensive and vague. Present the conclusions punctually and on the proposed objectives, on the other hand, include part of the conclusions made in the discussion of the results.

**Response 4:** The authors are extremely thankful to the reviewer for this thoughtful point. The authors completely agree with you, and we added it, kindly check the updated paper (Conclusions section).

**Reviewer 2:**

**Comments to the Authors:**

**Comment**:** After evaluating the manuscript titled "Advanced Design and Engineering Economical Evaluation of a Low-Cost Automatic Sugar-cane Seed Cutting Machine Based RGB Color Sensor and IoT Technology" my decision is: Major revision.

Overview: I acknowledge the importance of the topic covered in the manuscript and the development of intelligent mechanisms for sugar-cane seed cutting using sensors and IoT technology. However, the scientific contribution is limited. Therefore, if accepted for publication after the review process, I suggest it be considered as a technical article.

**Authors response**: The authors express gratitude to the reviewer for their encouragement and trust in our work. However, this paper is only a part of a larger project:**

**1.** [**https://doi.org/10.1371/journal.pone.0301294**](https://doi.org/10.1371/journal.pone.0301294)

**2.** [**https://aujes.journals.ekb.eg/article_345204_6670759135073095241fd79e475bc22f.pdf**](https://aujes.journals.ekb.eg/article_345204_6670759135073095241fd79e475bc22f.pdf)

**Comment-1: *Title: The title is lengthy and confusing. It needs modification.***

**Response-1:** The authors are extremely thankful to the reviewer for this thoughtful point. We completely agree with you, and we added it, kindly check the updated paper (paper title).

**Comment-2: *The abstract should present results in a way that allows first-time readers to grasp the content easily. Some abbreviations used are not explained for readers unfamiliar with them. Furthermore, the abstract should include a discussion of the results and conclude with findings and recommendations.***

**Response-2:** The authors are extremely thankful to the reviewer for this thoughtful point. The authors completely agree with you, and we added it, kindly check the updated paper (Abstract section).

**Comment-3: *Keywords: Avoid using terms already in the title.***

**Response-3:** The authors are extremely thankful to the reviewer for this thoughtful point. We agree with you, so we developed the keywords. Kindly check the updated paper (keywords).

**Comment-4: *The introduction is inadequate and does not contribute effectively to understanding the work. The justification lacks depth in convincing the reader of the research's relevance. The problem is not clearly presented, making it difficult to understand the significance of this work. Specifically emphasize how developing an automatic sugar-cane seed cutting machine using color sensors and IoT can address identified challenges like improving efficiency and reducing operational costs. Additionally, the study's hypothesis is missing, hindering a comprehensive analysis of the manuscript's objectives. The manuscript's objectives need refinement, including specific aims. Furthermore, replace "[1]–[3]" and "[7]–[11]" with "[1–3]" and "[7–11]" throughout the manuscript. Update the citation "(Statista, 2018)" to [4] in line 55 with current data on sugar-cane production. Update reference [5] for the same reason in line 56.***

**Response-4:** The authors are extremely thankful to the reviewer for this thoughtful point. We completely agree with you, and we adjusted and developed the introduction section, kindly check the updated paper (Introduction section).

**Comment-5: *The methodology is incomplete and needs detailed explanations of proposed procedures. More information is required for readers to replicate the experiment.***

**Response-5:** The authors are extremely thankful to the reviewer for this thoughtful point. We completely agree with you, and we added it, kindly check the updated paper (Title 2.5).

**Comment-6: *Could the authors provide more details on specific procedures used for sensor calibration? For instance, what criteria were used to select reference colors, and what were the lighting conditions during calibration? Was there a validation process for sensor accuracy?***

**Response-6:** The authors are extremely thankful to the reviewer for this thoughtful point. We completely agree with you, and we added it, kindly check the updated paper (Title 2.4 & subtitles 2.4.1. and 2.4.2.).

**Comment-7: *How was sensor reliability in detecting and counting sugar-cane characteristics determined.***

**Response-7:** The authors are extremely thankful to the reviewer for this thoughtful point. But the Ultrasonic sensor was used only for counting the number of sugarcane seeds after the cutting process, the operating principle is shown in subtitles 2.2.4 and 2.3.2. & Figures 8 and 12. kindly check the updated paper (paper title).

**Comment-8: *A more detailed description of performance tests evaluating the machine's effectiveness would be beneficial. For example, how were cutting tests conducted for different stem diameters and working conditions? Were tests repeated for result consistency? Was reproducibility analyzed to ensure data consistency? What statistical methods were used to analyze test data? Statistical metrics evaluating accuracy and precision are necessary. Could the authors elaborate on these metrics?***

**Response-8:** The authors are extremely thankful to the reviewer for this thoughtful point.

- We used the developed machine for cutting process, However, this paper is only a part of a larger project:

1. <https://doi.org/10.1371/journal.pone.0301294>

2. <https://aujes.journals.ekb.eg/article_345204_6670759135073095241fd79e475bc22f.pdf>

Where the performance tests of the ASSCM were conducted under laboratory conditions in the Department of Agricultural Engineering, Aswan University, kindly check the updated paper (subtitle 2.5.).

- All calibration tests were replicated at least three times, kindly check the updated paper (subtitle 2.4.1. & 2.4.2.).
- We used many statistical analysis and programs for analysis the obtained data, kindly check the updated paper (subtitle 2.7. & 3.1.2. & 3.2.).

**Comment-9: *How was sensor reliability in detecting and counting sugar-cane characteristics determined.***

**Response-9:** This paper is a part of a larger project, and most of these points have been demonstrated in the current publications.

1. <https://doi.org/10.1371/journal.pone.0301294>

2. <https://aujes.journals.ekb.eg/article_345204_6670759135073095241fd79e475bc22f.pdf>

**Comment-10: *Results and Discussion: Results are well-presented but lack discussion and scientific justification. Weaknesses in the research are not addressed. For instance, manual procedures are still required despite machine use. Comparisons with relevant prior studies should also be included. This can contextualize results within the broader field and highlight unique research contributions. How do the economic analysis results demonstrate the proposed system's feasibility and potential benefits? Future research suggestions based on current findings should also be included.***

**Response-10:** The authors are extremely thankful to the reviewer for this thoughtful point. We completely agree with you, and we adjusted and developed the Results and Discussion section, kindly check the updated paper (Results and Discussion section). Where the comparison of the ASSCM with other technologies has been demonstrated in subtitle 3.6. and table 4. In addition, Future research suggestions based on current findings has been demonstrated in future works in conclusion section.

**Comment-11: *Conclusions: Authors should address study objectives in this section.***

**Response-11:** The authors are extremely thankful to the reviewer for this thoughtful point. We completely agree with you, and we adjusted and developed the Conclusions section, kindly check the updated paper (Conclusions section).

**The authors once again thank the learned Editors and Reviewers for their valuable comments for improving the quality of the manuscript.**
